# Bioactive Lyocell Fibers with Inherent Antibacterial, Antiviral and Antifungal Properties

**DOI:** 10.3390/molecules29092054

**Published:** 2024-04-29

**Authors:** Frank Wendler, Thomas Schulze, Janine Bauer, Benjamin Redlingshöfer

**Affiliations:** 1 Smartpolymer GmbH, 07407 Rudolstadt, Germany; 2 Centre of Excellence for Polysaccharide Research, Thuringian Institute of Textile and Plastics Research e.V., European Polysaccharide Network of Excellence (EPNOE), 07407 Rudolstadt, Germany

**Keywords:** cellulose fiber, lyocell fiber, bioactive fibers, antibacterial, antiviral, antifungal

## Abstract

Functional Lyocell fibers gain interest in garments and technical textiles, especially when equipped with inherently bioactive features. In this study, Lyocell fibers are modified with an ion exchange resin and subsequently loaded with copper (Cu) ions. The modified Lyocell process enables high amounts of the resin additive (>10%) through intensive dispersion and subsequently, high uptake of 2.7% Cu throughout the whole cross-section of the fiber. Fixation by Na_2_CO_3_ increases the washing and dyeing resistance considerably. Cu content after dyeing compared to the original fiber value amounts to approx. 65% for reactive, 75% for direct, and 77% for HT dyeing, respectively. Even after 50 household washes, a recovery of 43% for reactive, 47% for direct and 26% for HT dyeing is proved. XRD measurements reveal ionic bonding of Cu fixation inside the cellulose/ion exchange resin composite. A combination of the fixation process with a change in Cu valence state by glucose/NaOH leads to the formation of Cu_2_O crystallites, which is proved by XRD. Cu fiber shows a strong antibacterial effect against *Staphylococcus aureus* and *Klebsiella pneumonia* bacteria, even after 50 household washing cycles of both >5 log CFU. In nonwoven blends with a share of only 6% Cu fiber, a strong antimicrobial (CFU > log 5) and full antiviral effectiveness (>log 4) was received even after 50 washing cycles. Time-dependent measurements already show strong antiviral behavior after 30 s. Further, the fibers show an increased die off of the fungal isolate Candida auris with CFU log 4.4, and nonwovens made from 6% Cu fiber share a CFU log of 1.7. Findings of the study predestines the fiber for advanced textile processing and applications in areas with high germ loads.

## 1. Introduction

Cellulose represents the most abundant biopolymer, whose unique properties make it an ideal material for many different applications above its original role as a common textile fibre [1]. Thus, it is receiving more and more attraction, especially in view of resource shortening, sustainability and the replacement of oil-based raw materials.

In combination with environmentally friendly processing technologies, such as the Lyocell process, we can observe its role as a versatile material of the future, especially as technology is able to process raw materials of natural origin (wood, cotton, hemp) and/or *end-of-life* textiles. As a beneficial feature, the fibrous products can also have functional properties. In addition to wear comfort, durability and fashionable chic, a garment should also regulate moisture, act thermoregulatively or have an antimicrobial effect.

Textiles bearing bioactive properties are known to possess protection capabilities against harmful bacteria, viruses or fungi. More recently, the COVID-19 pandemic has led to the awareness of how fast the sudden occurrence of viruses causing highly contagious viral diseases such as influenza, but also rabies, or hepatitis, makes special protection measures inevitable. Furthermore, besides effective vaccination protection, wearing mouth and nose masks has turned out to be a rather simple but quite effective measure to limit retransmission. Some manufacturers equip these masks, which are primarily intended to prevent the spread of aerosols, with an active ingredient such as silver (Ag) or copper (Cu) [2] to devitalize viral pathogens quickly.

Although Ag is still widely used due its favorable broadband antimicrobial effect, its environmental impact, especially the distinct fish toxicity, is increasingly arousing suspicion. For this reason, and due to the lack of less expensive alternatives, there is an urgent need to keep leaching effects at washing as low as possible, while ensuring such a concentration at the fiber surface which guarantees an optimal efficiency. Since Ag needs a wet environment to develop its antimicrobial activity, an increased surface area, such as that of nanoparticles, is expected to support ion release, leading to an increased antibacterial efficiency [3], even under common ambient conditions. In addition to this, Ag may also possess effective antiviral qualities.

Meister et al. demonstrated the significant differences in antiviral properties of Ag and Cu. To inactivate SARS-CoV-2 viruses, the silver concentration needs to be tenfold higher. It has further been established that bacteria rather than viruses are much more Ag-sensitive to metabolic processes such as energy generation or cell proliferation [4].

In ancient times, Cu was used to sterilize chest wounds and drinking water. Unlike Ag, it is an essential trace element and, therefore, is harmless to human beings [5,6]. Cu is responsible for the formation of enzymes, which control the mineralization of bones, regulation of neurotransmitters, heart functions, immune mechanisms, iron metabolism, etc. Cu and Cu alloys possess strong antibacterial properties against Gram-positive and Gram-negative bacteria [7]. The inactivation of bacteria occurs when in contact with the surface, which happens in a dry state as well [8]. Active ingredient Cu does not wear out over the period of use, and it is effective even on dirty surfaces [7].

Beyond that, the virucidal properties of Cu surfaces and coatings are intensively studied. A review by Rakowska et al. is available [9]. Both Cu(I) and Cu(II) ions are active in virus inactivation. As described by Warnes [10], Cu(II) is mainly released from the surface, which can be reduced to Cu(I) under certain conditions. When supported by reactive oxygen species (ROS) from cell debris, highly reactive hydroxyl radicals are then formed via Fenton’s reaction (Equations (1)–(3)).

2Cu^+^ + 2O_2_ (aq) → 2Cu^2+^ + 2O_2_^−^ (1)

2O_2_^−^ + 2H^+^ → H_2_O_2_ + O_2_ (2)

Cu^−^ + H_2_O_2_ → Cu^2+^ + OH^−^ + OH˙ (3)

Cu ions and free radicals are able to attack enveloped viruses and damage their protein shells and membranes. Even in communal areas, Cu helps reduce the retransmission of respiratory viruses like COVID-19.

Generally, the survivability of viruses depends on the porosity and hydrophobicity of the attacked surface and surrounding prevailing ambient conditions like temperature, pH value and humidity, and the presence of reactive species. All of these factors have emerged during evolution to stabilize and protect them from any damage [9]. Thus, it is necessary to start fighting against both non-enveloped viruses like Rhinovirus, and enveloped viruses such as Ebola, Zika, Influenza or COVID-19.

Textile materials may have pores, giving them large enough surface areas to offer bacteria and viruses ideal colonization sites. Usually, when in daily use, frequent contact with other surfaces like skin and seating furniture as well as the adsorption of aerosols, fats, and proteins makes the deposition of all kind of germs very likely. Bacteria and viruses may persist for a duration of days to months [11]. This fact has to be at least taken into account for garments, but is indispensable for hospital clothing.

In [12], the immobilization of copper by the impregnation of fibers with Cu(II)-salts, the subsequent reduction to Cu(I) or Cu, and the deposition of colloidal copper solutions or nanoparticles are reported. Immobilizing copper species in the initial steps of spinning processes (prior to or during spin mass preparation) is less useful due to prevailing conditions that may lead to premature conversion, precipitation or leaching. This is especially valid in the case of Lyocell processes, where the solvent itself can act as a distinct oxidizer [13,14].

Thus, it was the objective of the present study to incorporate Cu ions in a Lyocell fiber, to achieve a long-lasting antibacterial permanence and to achieve antiviral and antifungal effects. The results are expected to significantly improve the performance of bioactive textiles and nonwovens in the future.

## 2. Results and Discussion

### 2.1. Permanent Agent Incorporation

**Figure 1:**
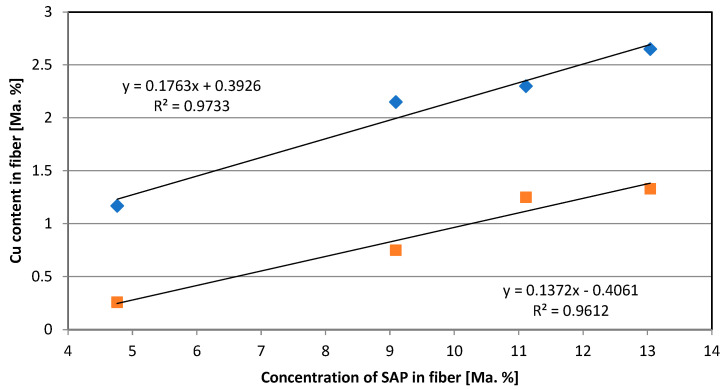
Relationship between SAP concentration in fiber and Cu content in the fiber directly after fiber production [**◊**] and after 20 washing cycles [**☐**].

Fibers were produced with varying SAP concentrations and loaded with the same CuSO_4_·5H_2_O solution following the same procedure (concentration, time, temperature). Cu was measured in dried fibers directly after production and after 20 washing cycles (Figure 1). The loss after washing can be estimated to approx. 40%. Both series of measurements follow a linear dependence. Cu binding is influenced only by SAP concentration. Diffusion effects, the accessibility of the COO^−^ groups of SAP inside the cellulose skeleton and the exchange with existing sodium (Na) ions bound to SAP have no impact.

When considering a Cu value of 2.65% of the original fiber, 0.834 mmol COO^−^/g with divalent Cu can be calculated, as listed in Table 1. With the same loading procedure, pure SAP was treated with CuSO_4_·5H_2_O solution, which revealed a value of 0.615 mmol COO^−^/g. Under consideration of the testing uncertainty and deviation in technical fiber production with loading/fixation, a similar range of COO^−^ values can be obtained. It can be concluded that Na is partly displaced by Cu.

**Figure 2:**
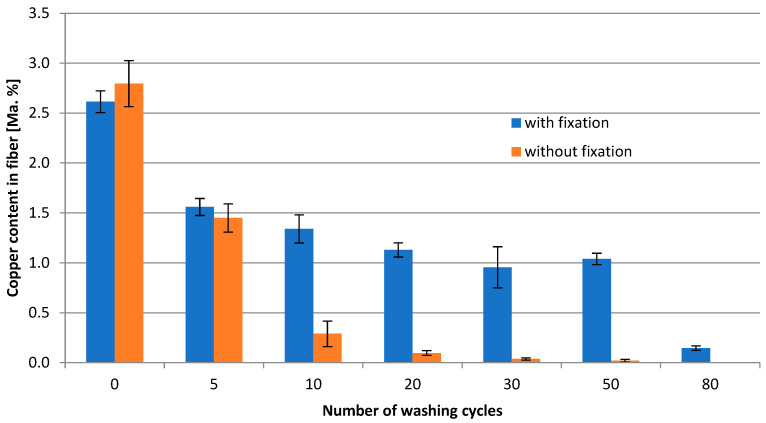
Relationship between washing cycles and Cu content in the fiber directly without [**☐**] and with Na_2_CO_3_ fixation [**◊**].

To investigate the necessity of Cu ion fixation inside the fiber, washing trials were conducted with a loaded fiber without Na_2_CO_3_ fixation and compared to a fiber with Na_2_CO_3_ fixation. Figure 2 represents the Cu content of both fibers after 50 washing cycles. After 10 washing cycles, the fiber without fixation already lost approx. 90% of the original Cu content, and after 50 washing cycles, only traces of Cu could be detected. In contrast, the fixation ensures a loss of only approx. 50% after 10 cycles and 60% after 50 washing cycles (significant difference according to *t*-test; *p* = 0.95; f = 4). A total of 50 washing cycles is usually applied to simulate the lifespan of a textile fabric. Apart from that, the maintenance of a function (agent) inside textiles is challenging in view of depot and release mechanisms.

To take it further, the fiber with fixation was treated with 80 washing cycles, resulting in a residue of 10% of the origin Cu content. Even after 120 cycles, ca. 8% of Cu could be found, and after 160 washing cycles (not shown in Figure 2) 5% of Cu could be found.

### 2.2. Dyeing Experiments

**Figure 3:**
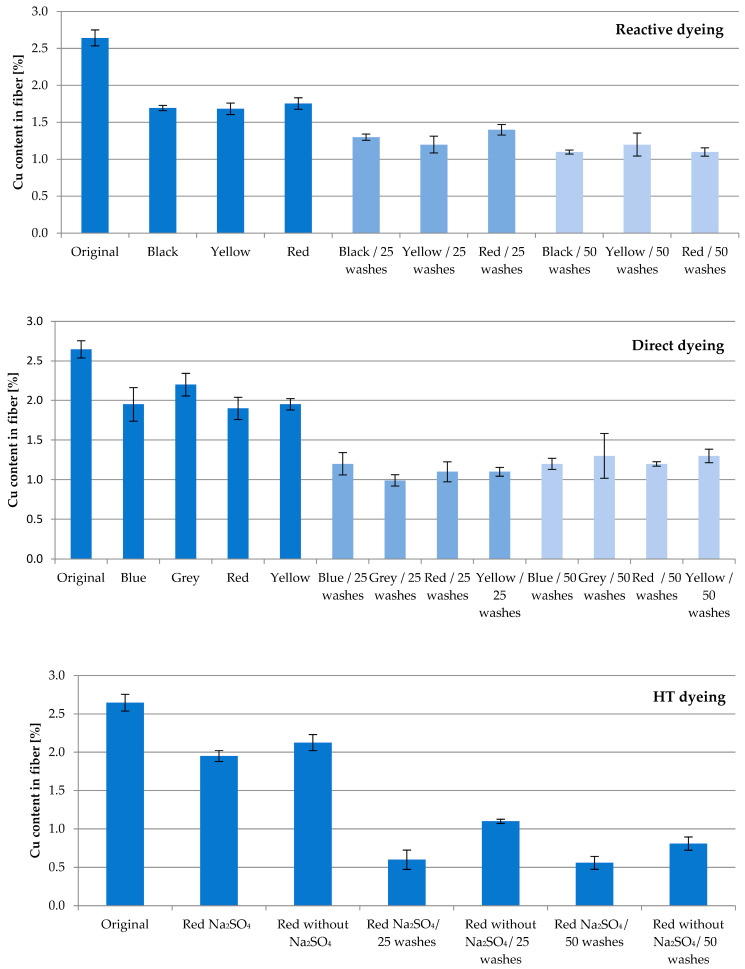
Relationship between washing cycles and Cu content in the fiber for reactive, direct and HT dyeing procedures using different colors.

Fiber with 13.04% SAP loaded with Cu underwent several dyeing attempts. The aim of this study was to investigate the washing permanence combined with textile processing under harsh conditions. The fibers were subjected to direct, reactive and high-temperature (HT) dyeing processes, which led to an expected decrease in the active ingredient concentration. The Cu content of the fibers after dyeing compared to the original value of 2.65% amounted to approx. 65% for reactive, 75% for direct, and 77% for HT dyeing, respectively (Figure 3). The obvious differences to the corresponding original value were confirmed according to *t*-test (*p* = 0.95; f = 1).

Subsequent household washes resulted in further losses. Whereas the Cu content after 25 washing cycles averaged 49% for reactive and 41% for direct dyeing, HT dyeing resulted in higher losses of Cu, resulting in 32%. Compared to reactive and direct dyeing, the HT process conditions of a high temperature and pressure exhibit an increased challenge in fiber processing.

After 50 washing cycles, 43% of the original Cu content for reactive, 47% for direct and 26% for HT dyeing are found. No significant difference was calculated between the results of 25 and 50 washing cycles for all three dyeing processes (according to *t*-test; *p* = 0.95; f = 1–3). This result reflects the very tightly bound Cu ions inside the cellulose–acrylate network.

Unexpectedly, Glauber’s salt Na_2_SO_4_ in HT dyeing minimizes the recovery of Cu. Used to enhance the absorption ability of dyestuffs in general, at an applied temperature of 130 °C in HT dyeing, Na_2_SO_4_ potentially displaces Cu ions. Further studies on this matter will follow.

**Figure 4:**
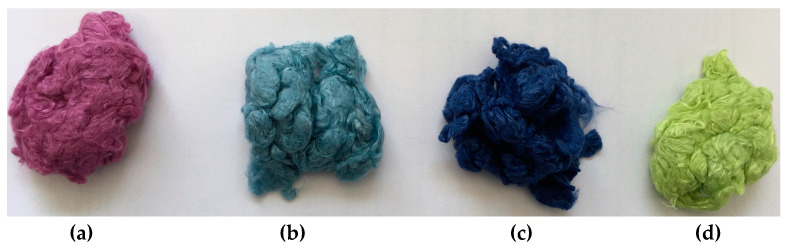
Color shades after direct dyeing. Target colors: red (**a**), grey (**b**), blue (**c**), yellow (**d**).

As an example, Figure 4 shows the resulting color shades after direct dyeing processes. The blue color of the original fiber prevails, and the shade of the color changes as expected.

### 2.3. X-ray Diffraction (XRD) Studies

Since even hydrophilic polymer matrices are not expected to permanently immobilize any type of ions being introduced by, for instance, water-soluble ingredients, an additional fixation step is always necessary. Such a treatment usually leads to a localized precipitation of an insoluble compound (e.g., halogenide, hydroxides). Interestingly, our studies revealed the occurrence of subordinated reactions in the fiber, where either the projected precipitation form does not develop or more preferably, the cation changes its state of oxidation.

**Figure 5:**
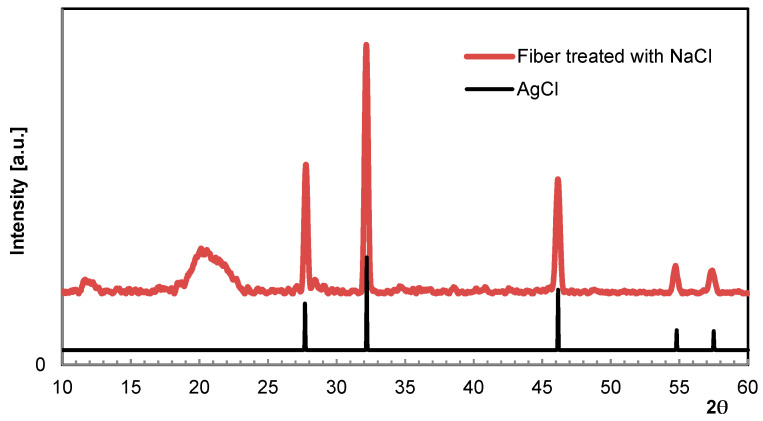
WAXS scan of Ag fiber treated with NaCl (spectrum above) in comparison to AgCl (spectrum below, database [18]).

As starting point for these investigations, a previous study should be referenced here to introduce the reliability of XRD measurements [15,16]. In order to immobilize Ag ions permanently, AgNO_3_-loaded fibers were allowed to become remoistened in a NaCl solution, resulting in AgCl precipitation. Although known to be insoluble, the quantity of ions released either by the dissociation or formation of elementary Ag when exposed to light is sufficient for inducing a significant and long-lasting antibacterial effect. The presence and durability of AgCl crystallites in the fiber was verified by XRD [17]. Figure 5 shows the XRD scan of a fibrous specimen taken after a prolonged long storage period. In fact, the grey appearance of the fiber might be an indication of Ag separation, but no additional crystalline phases were found. Table 2 summarizes the scattering angle 2θ according to the Miller indices (hkl) available from the database [18].

**Figure 6:**
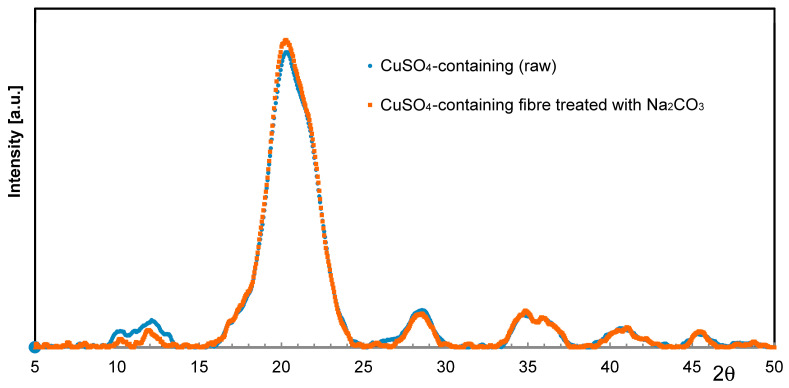
WAXS scan of an untreated Cu fiber (ο) and one treated with Na_2_CO_3_ (☐).

Considering the previous statements on Ag, especially in light of the fastness to washing, Cu immobilization is supposed to follow a similar reaction mechanism at precipitation. Rather than raising the pH value by caustic addition, soda might precipitate Cu inside the cellulose fiber matrix in a more efficient way. When treating Cu-loaded fibers with Na_2_CO_3_ solutions, slight color intensification is usually observed. However, no conceivable crystalline phases such as for instance Malachite (CuCO_3_·Cu(OH)_2_) or Posnjakite (Cu_4_[(OH)_6_|SO_4_]·H_2_O) could be detected by means of XRD (Figure 6).

This supports the assumption, that Cu ions are preferably bound to the carboxyl moieties of the incorporated acrylate polymer, and surplus Cu ions may exist in the form of less-ordered insoluble species in the cellulose–acrylate network at best.

**Figure 7:**
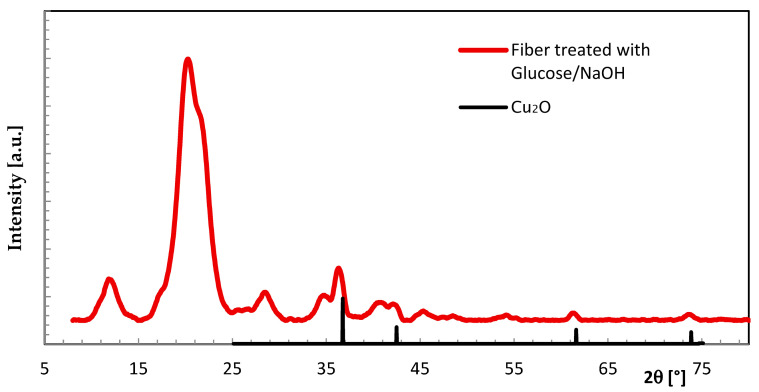
WAXS scan of Cu fiber treated with glucose/NaOH in comparison to Cu_2_O scan (database [18]).

Another route was taken with a combination of Cu fixation and valence change. The fiber loaded with CuSO_4_·5H_2_O was treated in a further step with glucose/NaOH. The formation of copper-(I)-oxide (Cu_2_O) is visually observed by a color change from blue to pale rose. In this case, the search for additional crystalline phases led to the detection of Cu_2_O. Even though the Cu concentration was rather low, the individual XRD signals were found to stand out from the cellulose background humps when comparing the data set with that of non-modified cellulose (Figure 7, Table 3).

### 2.4. Microbiological Effectiveness

#### 2.4.1. Antibacterial Effectiveness

**Figure 8:**
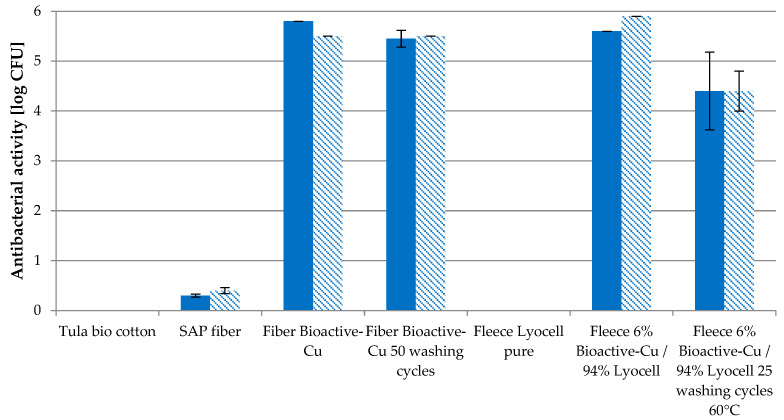
Antibacterial effectivity (log KBE) of Cu fiber and fleece (6% Cu fiber/94% Lyocell) against *Staphylococcus areus* (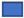) and *Klebsiella pneumoniae* (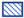) before and after washing.

Figure 8 displays the antibacterial effectivity of Cu fiber against Gram-positive *Staphylococcus aureus* and Gram-negative bacterium *Klebsiella pneumonia*. As referenced, Tula bio cotton, unloaded SAP fiber and pure Lyocell fleece were used to exclude the antimicrobial properties of other materials. Cu fiber shows a strong antibacterial effect, even after 50 household washing cycles of >5 log CFU. The reduction in Cu content after 50 washes by ca. 60% does not cause a reduction in antibacterial effectivity.

With only a 6% share of that fiber in a fleece with 94% pure Lyocell fiber, a strong effect was observed, even after 50 washing cycles.

#### 2.4.2. Antiviral Effectiveness

**Figure 9:**
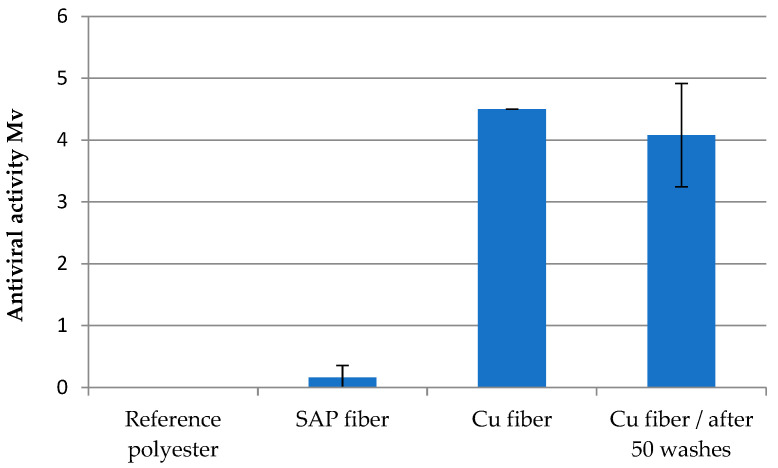
Antiviral effectivity (Mv) of Cu fiber before and after washing.

**Figure 10:**
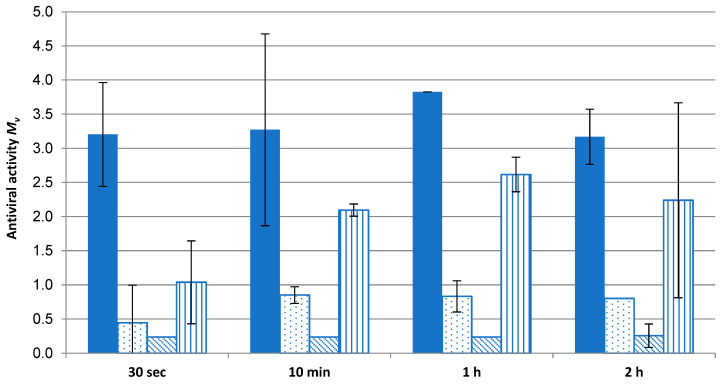
Relationship between time and antiviral effectivity (M_V_) of fleece (6% Cu_2_O fiber/94% Lyocell, 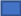) compared to commercial reference fleeces: 1 (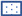), 2 (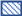) and 3 (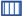).

Cu fiber fixed with Cu_2_O was used to investigate the antiviral effect against enveloped *bacteriphage phi6* viruses. Polyester and an unloaded SAP fiber were used as references. A pronounced antiviral efficacy was observed with a value of >log 4 for the original fiber as well as after 50 washing cycles (Figure 9, Table 4). Although the Cu concentration was reduced to 109 mg/kg, this amount ensureed efficient inactivation of the viruses. Prepared Cu_2_O fleece with a share of 6% Cu_2_O fiber and 94% Lyocell fiber showed an antiviral activity of >log 3 (Figure 10).

As the extent of virus fragmentation increases with contact time to allow access to the Cu ions and ROS to destroy the virus [10], antiviral effectivity was measured with respect to time. Figure 10 displays the extent of virus inactivation on a time scale. Even after 30 s, complete antiviral effectivity (>log 3) for the Cu_2_O fleece was observed. For comparison, three commercial fleeces used in face masks were tested for their antiviral effects over time. The antiviral agents of these masks are based on TiO_2_/AgCl adduct (fleeces 1 and 2) and polyhexamethylene biguanide (PHMB) (fleece 3). None of these fleeces reached the mark of log 3, which indicates complete effectivity. The significant difference of the values of the Cu_2_O fleece was confirmed according to *t*-test (*p* = 0.95; f = 1).

These results indicate the need for higher Ag ion concentrations compared to Cu ion concentrations to reach antiviral activity, as described by Meister et al. [4]. Even Cu compounds like Cu_2_O, Cu_2_S or CuCl retain their anti-infective properties [19] in comparison to Ag compounds. Only one competitor sample (fleece 3) can be evaluated as log 2 after 10 min. PHMB, mainly used to inhibit bacteria during wound healing, does not reach the antiviral effectivity of Cu. Wang et al. [20] reported that a PHMB-treated spandex fabric can kill 99% (log 2) of coronavirus within 2 h of contact.

#### 2.4.3. Antifungal Effectiveness

**Figure 11:**
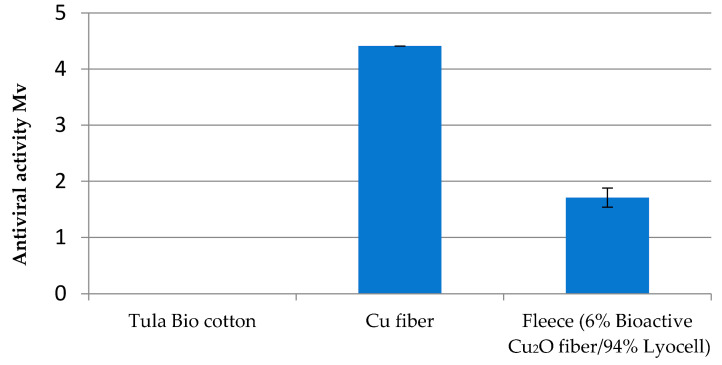
Antifungal effectivity (log KBE) of Cu fiber and fleece (6% Cu fiber/94% Lyocell) against *Candida auris*.

As there are indications that fungi have mechanisms to tolerate metal ions including entrapment within cell wall components [21], it was challenging to expand the test regime of Cu fiber for antifungal effectiveness. To limit the choice of promising fungi species, this study concentrated on ubiquitous and simultaneously disease-causing types. The growth of *Aspergillus fumigatus* DSM 819 and *Chaetomium globosum* DSM 1962 on the top and bottom and in underlying agar medium was not reduced by Cu fiber. The resilience of Aspergillus species against Cu surfaces was described by Weaver et al. [22]. However, a strong effectivity of 4.4 log CFU against *Candida auris* has been proved for Cu fiber. The fleece with a 6% share of Cu fiber with 94% pure Lyocell fiber shows a weak effectivity of at least 1.7 log KBE (Figure 11).

## 3. Conclusions

The equipment of Lyocell fibers with bioactive features was described and discussed in the context of effectivity against bacteria, viruses and fungi. Bioactive Cu fiber is produced in a three-step process. Firstly, a high amount of polyacrylate polymer (>10%) is homogeneously distributed in a spinning solution of cellulose and solvent. The basic requirements are a very small particle size of the additive and intensive mixing of the spinning solution. Only by this way can the additive be firmly incorporated into the fibrillar structure of cellulose during the dry/wet spinning process and evenly distributed over the entire fiber cross-section. Subsequently, the fiber is treated with a solution of a metal salt, CuSO_4_, thus finally fixing the metal ion in it.

Variation in polyacrylate concentration leads to a linear correlation of Cu concentrations in the fiber of up to 2.65%, which continues after 20 household washing cycles with the same linear correlation. As the loss after washing amounts to approx. 40% over the whole Cu concentration range, the binding of Cu is only influenced by the amount of polyacrylate in the fiber. Apart from washing resistance, fixation by Na_2_CO_3_ ensures an advantageous dyeing behavior. Cu concentrations compared to the original fiber value of approx. 65% for reactive, 75% for direct, and 77% for HT dyeing, respectively, are measured. Further treatment of dyed fibers with 50 washing cycles reveal recoveries of 43% for reactive, 47% for direct, and 26% for HT dyeing. The XRD technique was used to study the binding characteristics of Cu inside the fiber. After fixation, Cu(II) is ionically bound inside the cellulose/ion exchange resin composite. The reduction of Cu(II) to Cu(I) during the fixation process with glucose/NaOH gives rise to the presence of Cu_2_O crystallites in the fiber.

The bioactivity of Cu against *Staphylococcus aureus* and *Klebsiella pneumonia* bacteria is proved, with a >log 5 CFU, even after 50 household washing cycles. Nonwoven blends with a share of only 6% Cu fiber show a strong antimicrobial behaviour of >log 5 CFU and >log 4 CFU after 25 washing cycles. The antiviral effectiveness of the Cu fiber before and after 50 washing cycles amounts to >log 4. The blend with 6% Cu fiber is measured as >log 3 and is quite effective after just 30 s. Furthermore, the Cu fiber shows an inactivation of the fungal isolate Candida auris with a CFU log 4.4, and nonwovens made from 6% Cu fiber had a CFU log 1.7.

This developed three-step technology, in contrast to finishes on the fiber surface, leads to a strong binding of the metal ion to the innermost layers of the fiber and at the same time, allows for the adjustment of the active ingredient concentration (% range). Consequently, the controlled migration (sub-micro range) of the active ingredient to the surface is adjustable. This integrated depot/release function enables a high washing permanence and textile processing, even under harsh conditions.

The high active ingredient efficiency allows for the use of small proportions (2–6%) in the textile composite, as shown in Figure 8 for a needle felt fleece with 6% Cu fiber content. As a result, the environmental impact is significantly reduced, while cost efficiency increases due to longer efficacy with the same additive input.

This study shows that Lyocell fibers can be modified with a low amount of additives like ion exchange resins. After loading with copper and fixing, there are many promising applications: everyday clothing, outdoor textiles, hospital textiles sector, home textiles or fleeces for face masks, and technical applications like air-conditioning systems or upholstery.

Bioactive fibers are gaining more interest in functionalizing textiles for garments and technical applications. Textiles with an integrated bioactive function can contribute to stopping waves of infections. Whereas high effectivity of the function is preferably targeted in most studies, the permanence of the function appears as a secondary concern. A long-lasting effect can have benefits such as saving resources, reducing textile waste, and minimizing the release of ingredients into environment.

Staple fibers can be produced in counts from 2.3 to 6.7 dtex, the finer of which are suitable for yarn counts up to Ne 30. This results in an application spectrum ranging from textiles in the hospital sector to nonwovens for filters.

## 4. Materials and Methods

### 4.1. Materials

A spruce sulfite pulp with DP 590 from Domsjö fabriker AB, Domsjö/Sweden, *N*-methylmorpholine-*N*-oxide of BASF/Leverkusen/Germany as a 50 wt.% aqueous solution, and polyacrylate from Evonik/Essen/Germany were used to produce the SAP fiber. SAP is partly crosslinked with NaOH, containing 3.43 mmol COO^−^/g (Titration) and 19.9% Na (ICP-OES). Glucose, AgNO_3_, NaCl, CuSO_4_·5H_2_O, and Na_2_CO_3_ were purchased from VWR International GmbH/Darmstadt/Germany as p.a. products.

### 4.2. Preparation of Modified Lyocell Staple Fibers and Nonwovens Thereof

Lyocell staple fibers 38 mm in length were produced according to [23] with the following SAP concentrations in the fiber: 4.76%, 9.09%, 11.11%, and 13.04%. In total, 1 kg of SAP fibers were soaked in 20 L deionized H_2_O and loaded with 0.15 M CuSO_4_·5H_2_O solution. After 20 min, with intensive stirring, the fibers were spun-off and centrifuged. In a second treatment bath, the fibers were softened at 40 °C using a common finisher, e.g., Avilan R.A. of Archroma. Fixation occurred directly after Cu loading with 10 g/L Na_2_CO_3_ solution, and it was stirred there for 20 min. An amount of 10 g/L glucose solution at pH 11 (NaOH) was used for fixation with valence change. In a similar way, Ag fibers were prepared using AgNO_3_ for loading and NaCl as fixation treatment.

Nonwovens of ca. 200 g/m^2^ were prepared by needle felt preparation using 6% of Cu-loaded fiber with 94% pure Lyocell staple fiber.

### 4.3. Fiber Characterization

Cu fibers were characterized by fineness (DIN EN ISO 1973/DIN Media GmbH/Germany), tenacity and elongation at break, condition (DIN EN ISO 5079/DIN Media GmbH/Germany), and loop tenacity (DIN 53843/DIN Media GmbH/Germany): 6.9 dtex; 21.1 cN/tex; 9.8%; 5.2 cN/tex. Determination of Cu was carried out by ICP-OES according to DIN EN ISO 11885/DIN Media GmbH/Germany after microwave pressure digestion.

### 4.4. X-ray Diffraction (XRD)

XRD measurements were carried out using a D8-BRUKER diffractometer equipped with a position-sensitive detector, operated in normal transmission mode (θ/2θ-system) using copper radiation (λ = 1.54 Å, tube voltage: 40 kV, anode current: 40 mA). On the primary track, the beam divergence was limited by a 1 mm divergence slit after passing through a 2.5° axial Soller. The diffracted beam was then passed through a Ni filter and another 2.5° axial Soller before being detected. Specimens were prepared as flat pellets of 0.2 cm thickness with a constant density of 0.45 g/cm^3^ to ensure even volumes for the incident beam to pass through. Obtained data were smoothed by SAVITSKY-GOLAY-filtering, whereupon the background was subtracted in a way that left additional spectroscopic features that were not assignable to cellulose clearly visible.

### 4.5. Dyeing of Fibers

Direct dyeing was conducted using the following dyestuffs: Sirius Bordeaux 5B, Sirius Grey VGL, Sirius Blue BRR and Sirius Light yellow 5G. An amount of 1 g Cu fiber was dispersed in 50 mL deionized H_2_O, and Na_2_SO_4_ and 20 mg direct dye were added. The dyeing procedure occurred using an Ahiba Nuance coloring device for 60 min at 100 °C with 4 K/min and 50 U/min. After rinsing with H_2_O, the fibers were washed with 50 mL H_2_O in the device for 30 min at 40 °C anf 50 U/min and afterwards, were rinsed again with H_2_O and dried.

Reactive dyeing was carried out using the following dyestuffs: Brilliant yellow 4GL, Remazol black RL, Remazol brilliant red F3B. An amount of 1 g Cu fiber was dispersed in 10 mL deionized H_2_O, added with 20 mL dyeing solution containing 20 mg dye stuff and 1.5 g Na_2_SO_4_ and intensively stirred. The solution was heated up to 30 °C in a coloration device and held for 10 min at 30 °C. Then, 1 mL of 4% NaOH was added under stirring, heated up to 60 °C in the device, held at 60 °C, and cooled down to 40 °C. After rinsing under warm and cold H_2_O, the fibers were dried.

High-temperature dyeing was carried out with Remazol brilliant red F3B. An amount of 1 g Cu fiber was dispersed in 30 mL deionized H_2_O, adjusted with acetic acid to pH 4–5, stirred intensively and placed in the coloration device. After heating up to 130 °C and holding at 130 °C for 60 min, the beaker was cooled down to 40 °C. After rinsing under warm and cold H_2_O, the fibers were dried.

### 4.6. Microbiological Investigations

The antibacterial effect of the fibers was determined according to the test specification DIN EN ISO 20743/DIN Media GmbH/Germany “Textiles-Determination of the antibacterial effectiveness of textile products” by applying a defined amount of bacteria to the fibers in diluted nutrient solution and incubating them for 24 h at 37 °C. The bacteria were then removed by shaking 5 × 5 s, and the remaining number of surviving bacteria was determined using the plating method. From the logarithms of the bacterial cell numbers (CFU = colony forming units) obtained for a non-antibacterial sample (control material) and for the antibacterial fibers, the difference was calculated, which represents a measure of the antibacterial effectiveness, with a log 2 reduction meaning good antibacterial effectiveness and a log 3 reduction meaning very good effectiveness. Values > log 5.5 CFU reached the limit of determination of the procedure. Therefore, a standard deviation cannot be calculated.

The antiviral test was determined based on the test specification ISO 18184:2014/DIN Media GmbH/Germany “Textiles-Determination of the antiviral activity of textile products”. To this end, the enveloped bacteriophage phi6 was used as a surrogate virus for the human enveloped viruses Influenza A or SARSA-CoV-2 and applied to the fibers in a defined amount and incubated for 2 h at 25 °C. The phages were then removed by shaking, and the remaining number of surviving phages was determined using plaque titer assay. The difference, which represents a measure of the antiviral effectiveness, was calculated from the logarithms of the plaque titers (CFU = colony forming units) obtained for a non-antiviral sample (control material) and for the antiviral fibers, with a log 2 reduction meaning low antiviral effectiveness and a log 3 reduction indicating complete antiviral effectiveness. Values > log 4.5 CFU reached the limit of determination of the procedure. Therefore, a standard deviation cannot be calculated.

The antifungal testing effect of the fibers was determined according to the test DIN EN ISO 20743/DIN Media GmbH/Germany “Textiles–Determination of the antimycotical effectiveness of textile products” by applying a defined amount of yeast cell fungi to the fibers in diluted nutrient solution and incubating them for 24 h at 37 °C. The yeast fungi were then removed by shaking, and the remaining number of surviving fungi was determined using the plating method. From the logarithms of the bacterial cell numbers (CFU = colony forming units) obtained for a non-antifungal sample (control material) and for the antifungal fibers, the difference was calculated, which represents a measure of the antifungal effectiveness, with a log 2 reduction meaning good antifungal effectiveness and a log 3 reduction meaning very good effectiveness. Values > log 4.4 CFU reached the limit of determination of the procedure. Therefore, a standard deviation cannot be calculated.

## 5. Patent

Wendler, F.; Bauer, J.; Schulze, T. Wash-permanent bioactive cellulose fiber with antibacterial and antiviral properties. 2021, DE 102022109459 A1. Priority data 24 April 2021.

## Figures and Tables

**Table 1:** Cu loading approach.

	SAP–Cu Adduct	Cu Fiber (13.04% SAP)
Ma. % Cu	2.0	2.65
mmol Cu/g	0.315	0.415
mmol COO^−^/g	0.615	0.834

**Table 2:** WAXS data of Ag fiber treated with NaCl in comparison to database [18].

	(111)	(200)	(220)	(311)	(222)
AgCl (Database)	27.52°	31.88°	45.67°	54.13°	56.74°
Ag fiber treated with NaCl	27.7°	32.2°	46.2°	54.6°	57.5°

**Table 4:** Assessment criteria of antiviral effectivity acc. to ISO 18184:2014 (see Section 4).

	Effectivity
2.0 > *M_v_*	No antiviral effectivity
3.0 > *M_v_* > 2.0	Weak antiviral effectivity
*M_v_* ≥ 3.0	Complete antiviral effectivity

**Table 3:** WAXS data of Cu fiber treated with glucose/NaOH in comparison to database [18].

	(111)	(200)	(220)	(311)
Cu_2_O (Database)	36.46°	42.35°	61.41°	73.55°
Cu fiber treated with glucose/NaOH	36.1°	41.8°	61.5°	73.4°

## Data Availability

Data are available upon request.
